# Head Shape Heritability in the Hungarian Meadow Viper *Vipera ursinii rakosiensis*

**DOI:** 10.3390/ani13020322

**Published:** 2023-01-16

**Authors:** Duarte Oliveira, Bálint Halpern, Fernando Martínez-Freiría, Antigoni Kaliontzopoulou

**Affiliations:** 1 Independent Researcher, Travessa da Tapada 103, Landim, 4770-327 Vila Nova de Famalicão, Portugal; 2 MME BirdLife Hungary, Költő Str. 21, 1121 Budapest, Hungary; 3 Department of Systematic Zoology and Ecology, Institute of Biology, ELTE-Eötvös Loránd University, Pázmány Péter ave 1/C, 1117 Budapest, Hungary; 4 ELKH-ELTE-MTM Integrative Ecology Research Group, Pázmány Péter ave 1/C, 1117 Budapest, Hungary; 5 Doctoral School of Biology, Institute of Biology, ELTE-Eötvös Loránd University, Pázmány Péter ave 1/C, 1117 Budapest, Hungary; 6 CIBIO, Centro de Investigação em Biodiversidade e Recursos Genéticos, InBIO Laboratório Associado, Campus de Vairão, Universidade do Porto, 4485-661 Vairão, Portugal; 7 BIOPOLIS Program in Genomics, Biodiversity and Land Planning, CIBIO, Campus de Vairão, 4485-661 Vairão, Portugal; 8 Departament de Biologia Evolutiva, Ecologia i Ciències Ambientals, Facultat de Biologia, Universitat de Barcelona (UB), Av. Diagonal 645, 08028 Barcelona, Spain; 9 Institut de Recerca de la Biodiversitat (IRBio), Universitat de Barcelona (UB), Av. Diagonal 645, 08028 Barcelona, Spain

**Keywords:** geometric morphometrics, animal model, restricted maximum likelihood, maternal effects, paternal effects, additive genetic variance

## Abstract

**Simple Summary:**

Investigating how traits that are important for survival are inherited and influenced by other factors, such as parental quality and physical condition, is crucial for understanding their evolutionary potential, deciphering how they may contribute to local adaptation and taking such capacity into account for conserving threatened species. We studied head shape heritability in the Hungarian meadow viper to investigate the relative importance of inheritance and the maternal and paternal effects in driving the observed morphological variations. Our results show that offspring phenotypes are mainly determined by genetic factors and maternal effects, while paternal effects and residual environmental influences are minimal. This suggests a high evolutionary potential for head shape in the Hungarian meadow viper, which suggests a strong contribution of this ecologically relevant trait in shaping the ability of this endangered species to adapt to changing conditions and/or habitats and which would be useful to consider in captive breeding procedures for conservation efforts.

**Abstract:**

Understanding heritability patterns in functionally relevant traits is a cornerstone for evaluating their evolutionary potential and their role in local adaptation. In this study, we investigated patterns of heritability in the head shape of the Hungarian meadow viper (*Vipera ursinii rakosiensis*). To this end, we used geometric morphometric data from 12 families composed of 8 mothers, 6 fathers and 221 offspring, bred in captivity at the Hungarian Meadow Viper Conservation Centre (Hungary). We separately evaluated maternal and paternal contributions to the offspring phenotype, in addition to additive genetic effects, all determined using a mixed animal model. Our results indicate a strong genetic and maternal contribution to head shape variations. In contrast, the paternal effects—which are rarely evaluated in wild-ranging species—as well as residual environmental variance, were minimal. Overall, our results indicate a high evolutionary potential for head shape in the Hungarian meadow viper, which suggests a strong contribution of this ecologically important trait in shaping the ability of this endangered species to adapt to changing conditions and/or habitats. Furthermore, our results suggest that maternal phenotypes should be carefully considered when designing captive breeding parental pairs for reinforcing the adaptive capacity of threatened populations, whereas the paternal phenotypes seem less relevant.

## 1. Introduction

Natural selection drives adaptive processes by favoring individual phenotypes that work better, in terms of fitness, in certain environments and conditions (Darwin 1859). Traits that display heritable variance have adaptive potential in natural selection, which is crucial to the survival of populations across environmental gradients [1]. For populations to be able to adapt and survive the constantly changing conditions, the availability of genetic variations for fitness-related traits is a necessary cornerstone condition [2], as without such variations, the phenotype is not able to respond to selection [3]. Heritability measures the proportion of phenotypic variance determined by genetic variation [4], and it corresponds to the trait variability that parents can pass on to their offspring [5]. Therefore, measuring heritability is a very direct way of assessing the evolutionary potential of a trait [6,7]. Importantly, the evolutionary potential of populations, determined by the heritability of adaptively relevant traits, is essential for their capacity to survive and selectively modify their phenotypes under changing conditions [1,8].

Biological structures corresponding to different body parts are a remarkable aspect of an organism’s phenotype. They provide a connection between the genotype and the environment, and consequently, they frequently fulfil the aforementioned conditions for being adaptively relevant, exhibit ecologically driven variability and possibly include a strong heritable component [9]. The head of an animal is a very complex system that takes part in a diversity of functions, including prey capture and feeding, breathing, habitat use, predator avoidance, display and sensory perception, protection of the brain and sensory systems and intraspecific antagonistic interactions, to mention a few [10,11]. With all these functions, the head is in constant interaction with different elements of an organism’s environment, such as climatic conditions, structural complexity of the habitat or availability of food, and this raises the opportunity for the occurrence of strong selective processes. Indeed, numerous studies have demonstrated adaptive responses of head relative sizes and shape variations to environmental features, linking form, function and ecology in an ecomorphological framework (e.g., in Darwin’s finches [12], in fossorial lizards [13], in bats [14], in monitor lizards [15] and in aquatic snakes [16]). It comes, then, as no surprise that the relative head size and shape have also been shown to exhibit a strong heritable component and a high evolutionary potential in a wide array of vertebrates, including salamanders [17], lizards [4,5], mice [18], horses [19] and humans [20,21].

The lack of limbs in snakes makes them an outstanding system to study the evolutionary and ecological role of the head shape. Snakes frequently exhibit ontogenetic, sexual and phylogenetic variations in the head shape and dimensions relative to their body size [22,23,24]. Their frequently dimorphic heads have been linked to sexual selection acting through female choice or male–male interactions by combat or resource defense [24,25,26]. Snakes are also among the most remarkable vertebrates with respect to prey consumption; their suspended type of cranial articulation and their flexible jaws make them predators of extremely large prey relative to their body size, which, at the same time, constitutes an important ecological driver of their head size and shape evolution [26,27]. Habitat selection and lifestyle also have major influences on the evolution of snake head sizes and shapes; for instance, burrowing habits and fossoriality, which seem to be primitive in snakes, are associated with a small, compact and scarcely kinetic skull [28]. Finally, venomous and nonvenomous snakes differ in their head shapes and sizes mostly due to the presence of venom glands frequently located in a lateral position posterior to the eye and other modifications in the maxillary bones to bear large fangs [29].

Despite these patterns pointing to the high evolutionary potential of the snake head shape, the levels of heritability and genetically vs. parentally/environmentally determined variance sources have never been evaluated, to the best of our knowledge. Other snake phenotypic traits have been investigated for their genetic, maternal and environmental components of variation. Color and color pattern are the most studied traits, and they are known to exhibit a strong heritable component in several snake species [30,31,32], which has also been shown to be genetically correlated to—also heritable—antipredator behavior [30]. Reproductive traits are instead quite intriguing, where some are also strongly heritable while others have a practically null genetic component [33]. Similarly, the annual body condition [34] and immune physiology [33] seem to be mainly maternally determined. The single available study considering the heritability of head-related traits in snakes showed that head scales exhibit moderately high levels of additive genetic variations in a captively bred population of the Hungarian meadow viper (*Vipera ursinii rakosiensis*; family Viperidae) [35]. Despite the mainly taxonomic use of these traits [36], the results of that study may be indicative of the existence of a genetic component in the head morphological variance, which could also translate into a strong evolutionary potential for the head shape, as one would expect based on its ecological importance.

Based on this prediction, in this study, we used geometric morphometrics (GM [37,38]) to assess head shape heritability in the Hungarian meadow viper (*Vipera ursinii rakosiensis*) and investigate the relative importance of genetic, maternal, paternal and environmental components for determining phenotypic variance patterns in this ecologically relevant body trait. Addressing the heritable variations of characteristics with a potentially high adaptive value is interesting from an evolutionary perspective, but in this case, it can also provide an important tool for conservation. *V. ursinii* is one of the most endangered snakes in Europe, as it is considered Vulnerable (VU) on a global scale, due to the extreme fragmentation of its distribution range [39] and Endangered (EN) in the case of *V. ursinii rakosiensis*, due to population fragmentation and low overall population size across Hungary [40]. In this context, assessing its potential for adapting to changing conditions and/or new areas can be the key for guaranteeing the persistence or recovery of populations by ensuring a greater likelihood of survival when captive-bred animals (such as those used here; see below) are reintroduced to the wild.

Specifically, in this study, we address the following questions: (1) What is the strength and pattern of head shape heritability in *V. ursinii rakosiensis*? (2) What is the relative contribution of the maternal, paternal and environmental effects in generating its head shape diversity? By answering these questions, we aim to elucidate the genetic and external contributions to the head shape variations in vipers, increase our understanding of the evolutionary potential of this important phenotypic trait and contribute towards better practices for the conservation of this endangered viper species.

## 2. Methods

### 2.1. Specimens

The Hungarian Meadow Viper Conservation Programme, supported by the European Union’s LIFE fund, was built to protect the Hungarian meadow viper (*Vipera ursinii rakosiensis*) and provide means for habitat and population restoration in Hungary [41]. Since 2004, a captive breeding program conducted in the Hungarian Meadow Viper Conservation Centre has been breeding individuals in outdoor terraria to posteriorly release them to the wild [42,43]. This program is strictly controlled in terms of the captive management of breeding pairs and in terms of gestation conditions in such a way that clutches can be pooled across mothers, reproductive events and years, and therefore, it is receptible to a heritability analysis [41,42,43].

In this work, we analyzed head shape variations from photographs of 234 individuals bred and raised through this captive breeding program from 2004 to 2014 by capturing wild-ranging animals from two different geographic regions and, in continuation, crossed them in captivity, released them in the wild and followed them over the next few years. Our dataset included 12 families, composed of 8 mothers, 6 fathers and 221 offspring (Table S1).

### 2.2. Geometric Morphometrics

**Figure 1:**
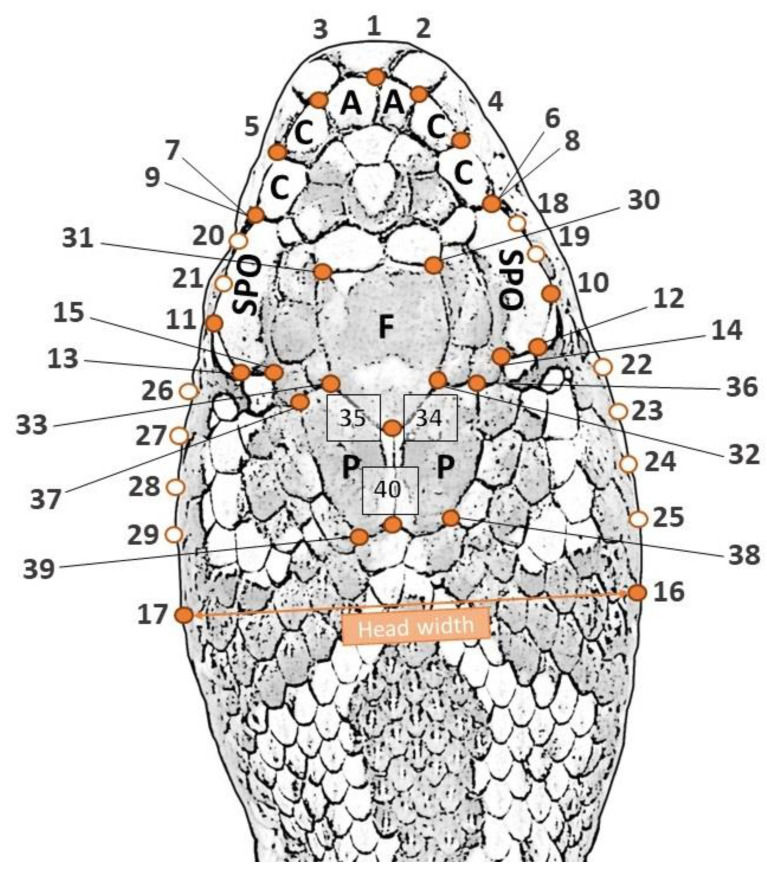
Position of landmarks (orange dots) and semi-landmarks (white-filled) used to quantify the head shape variation. A: Apical, C: Canthal, SPO: Supraocular (SPO), F: Frontal and P: Parietal scales. See Table 1 for detailed landmark descriptions.

To quantify the variations in head shape and size, we applied geometric morphometrics (GM) on high-resolution photographs of a dorsal view of the head. Twenty-eight fixed landmarks and twelve semi-landmarks were modified based on those previously used by [23] for *Vipera seoanei*. Specifically, fixed landmarks were added in the posterior and anterior corners of the frontal and parietal scales to provide more detail for the dorsal head shape and scalation variability, since these scales are rarely fragmented in *V. ursinii* (i.e., contrary to *V. seoanei*, for which these landmarks are not confidently available). These landmarks (Figure 1; Table 1) were digitized for each specimen using the program tpsDig2 [44], resulting in 40 pairs of x–y coordinates. After data acquisition, a Generalized Procrustes Analysis (GPA) [45,46] was applied in order to standardize the size, location and rotation effects and to preserve only the information related to shape, as implemented in the function *gpagen* of the geomorph R-package [47,48]. The positions of the semi-landmarks were optimized during the GPA superimposition by minimizing the bending energy [49]. This provided Procrustes residuals (shape variables) and an estimate of the size of individual landmark configurations represented by the centroid size (CS). Since asymmetry was not the focus of interest of this study, and it could affect the heritability inferences, a new perfectly symmetric landmark configuration was computed for each specimen by averaging the positions of all landmarks across the midline [4,50] using the function bilat.symmetry of the geomorph R-package. Finally, a principal component analysis (PCA) was carried out on the variance–covariance matrix of the landmark coordinates, and individual scores of the first 15 PCs were used as shape variables (due to technical limitations related to multivariate quantitative genetic analyses, i.e., in the maximum number of variables that VCE-6 can handle).

Before advancing to the heritability analyses, we tested for the presence of sexual dimorphism in adults and offspring separately. For this purpose, we used (M)ANOVA comparisons on the head shape variables and on log(CS) with sex as the predictor variable, as implemented through the function procD.lm of geomorph. The significance was tested using residual permutation procedures with 1000 repetitions [51,52]. As sexual dimorphism was not significant in either the adults or offspring (Table S2), we disregarded the sex of individuals from the heritability and variance component analyses to maximize the sample size.

### 2.3. Variance Components and Heritability of Head Shape

To determine whether the head shape is genetically inherited in *V. ursinii*, we used restricted maximum likelihood (REML) as implemented in VCE-6 to first estimate the genetic and phenotypic variance components. REML is the preferred method for estimating variance components from mixed effects models in quantitative genetics [53], and it is also appropriate for methods that yield multivariate data, such as geometric morphometrics [18,19]. One great benefit of using REML is that this method uses all the available information about relationships among the individuals included in the dataset, and it can easily accommodate unbalanced or nonstandard designs [53].

The particular nature of our data, which originated from captivity breeds of known parents, allowed us to separately evaluate maternal and paternal contributions to offspring phenotypes, in addition to additive genetic effects. As such, we used a complete version of the animal model that included, in addition to offspring identity for evaluating the genetic component of variance, mother and father identities as random effects. This provided the corresponding effects variance–covariance matrices, which allowed us to specifically estimate how much of the phenotypic variance was attributable to maternal and paternal contributions. Note that, here, because breeding was performed in highly controlled conditions, we may expect environmental and clutch effects unrelated to the quality of the parents to have a minimal contribution on the observed phenotypic variations. Finally, we also included log(CS) as a fixed effect to control for any potential allometric effects of size on the shape [4].

Once the animal model was fit through REML, we obtained four variance–covariance matrices corresponding to specific model effects of interest: the additive genetic (*G*), the maternal (*Mat*) and the paternal (*Pat*). Together with residual variance (*R*) typically attributable to environmental effects [54], these components sum up to the total phenotypic variance (*P*), i.e., *P = G + Mat + Pat + R*. Based on the total variance captured by each of these matrices, as represented by their traces, we could assess the relative contribution of each source in explaining the total phenotypic variance. We also examined the properties of these matrices, as expressed through their eigenvectors and eigenvalues, and projected the original shape variables back onto the calculated eigenvectors to visualize the shape variations related to each of them using deformation grids. To examine whether the total shape variation, as well as that explained by additive genetic and maternal effects (paternal effects had a minimal contribution; see Results), were aligned towards similar directions in the shape space, we calculated the angles between pairs of the first eigenvectors of each of them.

To estimate the shape heritability (*h^2^*), we used the breeders’ equation as generalized for multivariate data, i.e., $∆\bar{z}=GP^{-}S$ [2,55], $P^{-}$ being the generalized inverse. This allows one to calculate the matrix $GP^{-}$, which provides an estimate of heritability for multivariate data [56]. The principal eigenvector of $GP^{-}$ describes the genetic contributions to the phenotypic variance [19,57], and its eigenvalue summarizes the maximum additive heritability, *h^2^_max_* [58,59]. Similarly, we estimated the $MatP^{-}$ matrix to also examine the maternal contribution component to the phenotypic variance of the head shape and quantified the magnitude of these effects through the corresponding eigenvalues [4].

## 3. Results

The PCA conducted on the symmetric portion of the head shape variation provided a total of 38 components with positive eigenvalues, of which the first 2 summarized 56.79% of the total variance. The first 15 components, which were the data dimensions that VCE-6 can handle and were therefore analyzed to estimate the variance sources and heritability in the head shape of *Vipera ursinii*, cumulatively captured 95.69% of the total shape variance.

**Figure 2:**
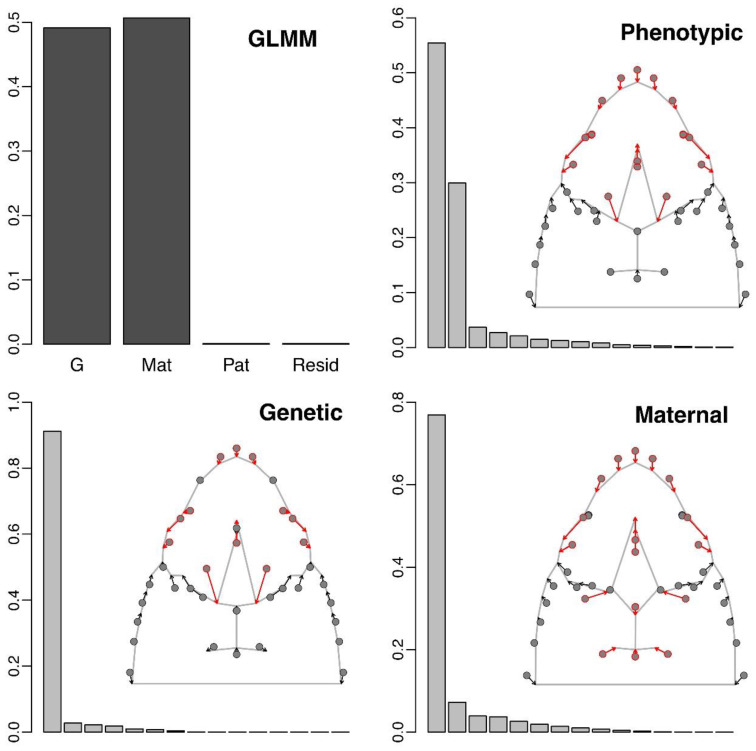
Relative importance of the additive genetic (*G*), maternal (*Mat*), paternal (*Pat*) and residual effects in explaining the total phenotypic variance in a GLMM (top left), as well as structure of the corresponding effect matrices visualized through the distribution of their relative eigenvalues. In all plots, the *y*-axis represents the proportion of variance explained (on a 0–1 scale). The arrows in red depict the highest variation of the phenotype from the three variance matrices.

**Figure 3:**
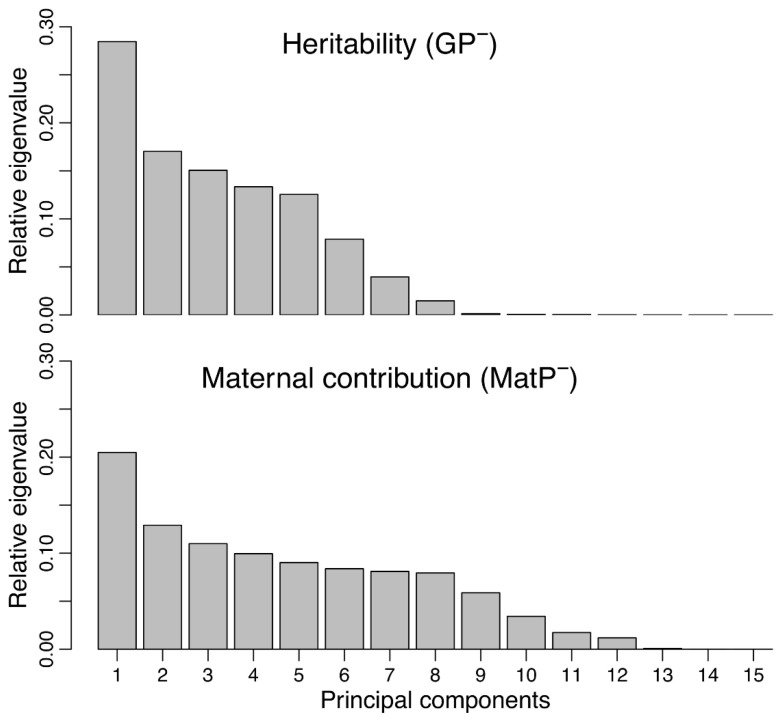
Eigenvalues of the matrices *GP^−^* and *MatP^−^*, which express the heritability and maternal contributions to the shape variations. The dominant eigenvalue describes the directions of the maximum additive heritability (*h*^2^) and maximum maternal effects, respectively.

The analysis of the relative contribution of the additive genetic, maternal and paternal effects in explaining the total phenotypic variance showed that these components represent 49.13%, 50.69% and 8.85 × 10^−4^%, respectively (Figure 2). That is, the additive genetic and maternal components together explained almost all of the phenotypic variance included in our dataset, with very similar contributions between them, while the contribution of the paternal effects was negligible and was not considered any further. Examination of the eigenvalues of the corresponding covariance matrices indicated that the total phenotypic variation is concentrated in a few dimensions, with the first six eigenvectors concentrating over 95% of the total variance (Figure 2). This effect was much more prominent for the genetic and maternal matrices, where the first eigenvector summarized 91.21% and 76.98% of the variance, respectively (Figure 2). Examination of the principal eigenvalues of the *GP^−^* and *MatP^−^* matrices indicated that the proportion of genetically inherited and maternally determined phenotypic variations was rather spread out through several shape dimensions, where the first five components cumulatively captured 86.43% and 63.31% of the variance, respectively (Figure 3).

The principal eigenvectors of the phenotypic (*P*) and additive genetic matrices (*G*) were fairly aligned with each other (vector angle θ = 29.29°), while the maternal effects (Mat) principal eigenvector exhibited substantially different directionality from both of them (θ = 43.23° and θ = 72.51° from the phenotypic and genetic eigenvectors, correspondingly). All three variance matrices captured a component strongly associated with the shortening of the snout, both through the posterior displacement of the front border of the head and the anterior displacement of the front edge of the frontal plate, as well as the posterior displacement of the ocular area of the head. However, global phenotypic and additive genetic variances also share a marked change of the shape of the frontal plate, with posterior displacement and narrowing of its posterior border, which translates into visible amplification of the eye entire area. Instead, the maternal effects were related to a forward displacement of the posterior border and a narrowing of the anterior border of the parietal scales.

## 4. Discussion

In this study, we investigated head shape heritability in the Hungarian meadow viper (*Vipera ursinii rakosiensis*), and we evaluated the relative contribution of the genetic, maternal, paternal and environmental components for determining the phenotypic variance patterns in this functionally and ecologically important body trait. The combination of geometric morphometrics—to quantify variations across families from a captive breeding program—and quantitative genetic approaches based on a full version of the animal model showed that the viper head shape is mainly determined by additive genetic and maternal effects, while the paternal and residual environmental contributions were negligible. This is the first study providing evidence for a large component of the heritable genetic variation in the head shape of vipers, highlighting the evolutionary potential of this ecologically relevant trait and facilitating the biological interpretation of the adaptive head shape variations in these organisms. Importantly, it suggests that this trait may be highly relevant for the capacity of *Vipera ursinii rakosiensis* to adapt to changing environments, which is key for the survival of such an endangered species and can be useful for informing and designing captive breeding programs in the context of conservation efforts.

Using the animal model, we evaluated the relative importance of different sources of head shape variations in *V. ursinii* and showed that it is dominated by additive genetic and maternal effects, which together explained a large part of the global phenotypic variance (Figure 2, top). Each of these effects was aligned with an unambiguous direction of the phenotypic space, as their dominant eigenvectors summarized 91.21% and 76.98% of their respective covariance matrices (Figure 2, bottom). Despite this strong directionality of the *G* and *Mat* matrices, the dominant eigenvalue of *GP^−^*, which represents the maximum heritability, was relatively low (*h_max_*^2^ = 0.29). Indeed, the heritable component of the global phenotypic variance was shown to be distributed through different directions (Figure 3, top), with 95% of the variance being spread out across the first six principal axes of *GP^−^*. The same was the case for the maternally determined component of the phenotypic variance, where the dominant eigenvalue of the *MatP^−^* matrix was 0.20, with 95% of the variance being spread out across the first 10 principal axes. These results suggest that, although the animal model clearly shows that the offspring head shape bears very high heritable and maternal contributions in this system, both these effects constitute to the complex shape responses that occur in different directions of the morphospace. From an evolutionary perspective, this suggests that *V. ursinii* has an additive genetic potential for producing evolutionary responses of different kinds without strong constraints on the variation, with different head parts relatively “free” to potentially vary independently [60]. This would confer high evolutionary flexibility [61], and it could be advantageous for adaptation to different environments [62] or changing conditions [63].

Further support towards the idea that the head shape additive genetic variance would probably enhance the head shape adaptability in *V. ursinii* comes from the comparison of the dominant eigenvectors of different covariance matrices. The relatively tight alignment of the additive genetic (*G*) with the global phenotypic (*P*) matrix suggests that the shape variations across individuals, which is the material “visible” to selective processes, is highly aligned with the available genetic variability and, therefore, heritable and potentially subject to selection [2]. This provides definite evidence that the head shape of vipers is a trait with genetic potential for responding to selection, which can be interpreted under different adaptive hypotheses on both the micro- and macroevolutionary scales [17,64]. The dominant eigenvectors of *P* and *G* are mainly related to a posterior displacement and narrowing of the posterior edge of the frontal plate, which causes the eye area to become proportionally enlarged (Figure 2). Head shape variations as a response to different biological factors has not been explored in *V. ursinii* to date using geometric morphometrics, limiting our capacity to interpret the specific areas of the head with a high additive genetic component. However, these areas partially correspond to shape variations across individuals of another species of the same genus, *V. seoanei*, sampled across different environmental conditions throughout its entire distribution range [23]. Whether these patterns are concordant across species of European vipers (genus *Vipera*), and to what extent they are actually also associated with processes such as local adaptation, within-species selective agents (e.g., sexual selection, diet variation etc.) and macroevolutionary differentiation across species remains to be examined. Hypothetically, an amplification of the eye area could be particularly relevant for a grass-dwelling species such as the Hungarian meadow viper, for instance, to increase light reception in environments with reduced luminosity [65] and to enhance binocular vision and their foraging capacity [66]. The high genetic diversity recently described across the distribution range of *V. ursinii rakosiensis* [67] suggests that there might be sufficient genetic variations available for within-species differentiation and local adaptation in the head shape in this endangered subspecies, but testing such a hypothesis would require a dense geographic sampling of the head shape morphology across different environmental conditions, which is quite complicated in this case given the scarcity of populations of this organism.

Interestingly, although comparisons across different systems are difficult, due to both biological and methodological reasons, these results are, in some cases, partially consistent but, in others, in contrast with results from previous studies that examined shape heritability in other species. While a high proportion of additive genetic variations for the head shape has been previously reported for both lizards [4] and salamanders [17], the maximum additive heritability (*h_max_*^2^) as captured by the eigenvalue of the first principal component of the *GP^−^* matrix was particularly high in salamanders, reaching over 0.8 in some populations. Instead, the heritable portion of the phenotypic variance was rather spread out over different shape directions for wall lizards [4], in accordance with what we observed here for the Hungarian meadow viper. Interestingly, the same is the case for the heritability of head scale counts in the same captive-bred population studied here, where moderate *h*^2^ values were reported for several head scalation characters studied univariately [35] (which makes *h*^2^ estimates higher by definition, though not directly comparable to the multivariate estimates used here). The relatively low heritability values that seem to characterize both the head shape and head scalation in this population of *Vipera ursinii rakosiensis* may be the outcome of both biological and methodological factors. First, dorsal head scalation has been circumstantially reported to vary ontogenetically in this species [68], which could mask part of the additive genetic proportion of the global phenotypic variance. Second, and most importantly, the high number of offspring (221) among a relatively reduced number of mothers (8) and even lower number of fathers (6) may partially bias the results reported in our study. While future studies would certainly benefit from amplifying the number of families examined here, this is the first time that both maternal and paternal identities have been combined to contrast their influence on the additive genetic and residual environmental effects of offspring phenotypes in such an endangered, wild-ranging, venomous snake.

From a conservation perspective, our results also highlight that the maternal effects, which usually reflect the maternal qualities and body conditions, have an equally strong and similarly multifaceted effect and should be carefully considered when designing captive breeding programs [69], as the ones from which our data were derived. Indeed, the paternal identity and residual environmental variance were minimal in our data, suggesting that, under controlled breeding conditions, it is mainly the maternal qualities that affects the offspring phenotypes. This matches the biological intuition for this viviparous snake under the breeding conditions that yielded our data. Indeed, in this case, the environmental effects on gestation, which are known to have a significant effect on offspring development [70], are expected to be minimized under laboratory-controlled conditions, especially for live-bearing organisms such as *V. ursinii*. However, it is important to note that the paternal contributions to offspring phenotypes—difficult as they are to identify with certainty in wild-ranging populations—could be higher in free-breeding animals in the field; this species is known to exhibit a biennial reproductive cycle, and females may mate every year but fecundation takes place when the body conditions are favorable, which usually occurs every second year, a fact that could lead to multiple paternities within a single clutch [71]. Similarly, variations in the body conditions across females in natural conditions (i.e., without ad libitum feeding resources, as those provided in the captive-breeding program), with a seasonal component, are common in vipers [72], a fact that could decrease the generally high maternal influence on the offspring phenotypes reported here.

## 5. Conclusions

Our study demonstrates that the head shape has major genetic and maternal contributions, with maternal effects being an important external contribution for the head shape diversity in this species. These results are important for the conservation of *V. ursinii rakosiensis*, since it is a species of high conservation concern, and this study shows that it has the potential to adapt to changing conditions. Studies using the animal model are required, since they are powerful tools to analyze the genetic parameters of the wild and captive breed populations, thus guaranteeing the survival and recovery of this population when reintroduced to the wild.

## Figures and Tables

**Table 1:** Descriptions of the position of landmarks (LM) and semi-landmarks (sLM) used to quantify the head shape in *Vipera ursinii*. Paired numbers represent landmarks at the right and left sides of the head, respectively.

Number	Description	Type
1	Anterior point between the two apical scales or anterior middle point of the single apical scale	LM
2, 3	External margin between the apical and the first canthal scale	LM
4, 5	External margin between the first and the second canthal scale	LM
6, 7	External posterior border of the second canthal	LM
8, 9	External anterior corner of the supraocular	LM
10, 11	External point of contact between the supraocular and the periocular	LM
12, 13	External posterior border of the supraocular, where it contacts with the posterior border of the periocular	LM
14, 15	Internal posterior border of the supraocular	LM
16, 17	External margins of the widest region of the head	LM
18–21	Outline of the supraocular scale (between LM 8–10 and 9–11)	sLM
22–29	Outline of the posterior head border (between LM 10–16 and 11–17)	sLM
30, 31	External anterior border of the frontal scale	LM
32, 33	External posterior border of the frontal scale	LM
34	Extreme external posterior border of the frontal scale	LM
35	Anterior point between the two parietal scales	LM
36, 37	External anterior border of the parietal scale	LM
38, 39	External posterior border of the parietal scale	LM
40	Extreme external posterior border of the two parietal scales	LM

## Data Availability

Landmark coordinates and specimen IDs used in this study will be available on GitHub upon publication.

## Supplementary Materials

The following supporting information can be downloaded at: https://www.mdpi.com/article/10.3390/ani13020322/s1: Table S1: Summary of the composition of the viper families analyzed. Table S2: Results of the (M)ANOVA comparisons implemented to test for sexual dimorphism in the head shape and log(CS) in the adults and offspring separately.

## References

[1] Hoffmann A.A., Merilä J. (1999). Heritable variation and evolution under favourable and unfavourable conditions. Trends. Ecol. Evol..

[2] Lande R. (1979). Quantitative genetic analysis of multivariate evolution, applied to brain: Body size allometry. Evolution.

[3] Monteiro L.R., Diniz-Filho J.A.F., Reis S.F., Araújo E.D. (2002). Geometric estimates of heritability in biological shape. Evolution.

[4] Sacchi R., Mangiacotti M., Scali S., Ghitti M., Bindolini B., Zuffi M.A.L. (2016). Genetic and phenotypic component in head shape of common wall lizard *Podarcis muralis*. Amphib. Reptil..

[5] Imhoff C., Giri F., Siroski P., Amavet P. (2018). Analysis of morphological variability and heritability in the head of the Argentine Black and White Tegu (*Salvator merianae*): Undisturbed vs. disturbed environments. Zoology.

[6] Lynch M., Walsh B. (1998). Genetics and Analysis of Quantitative Traits.

[7] Falconer D.S., Mackay T.F.C. (1996). Introduction to Quantitative Genetics.

[8] Frankham R., Ballou J.D., Briscoe D.A., McInnes K.H. (2002). Introduction to Conservation Genetics.

[9] Ricklefs R.E., Donald M., Wainwright P.C., Reilly S.M. (1994). Ecological and evolutionary inferences in morphology: An ecological perspective. Ecological Morphology: Integrative Organismal Biology.

[10] Kardong K.V. (2006). Vertebrates: Comparative Anatomy, Function, Evolution.

[11] Ziermann J.M., Diaz R.E., Diogo R. (2019). Heads, Jaws, and Muscles: Anatomical, Functional, and Developmental Diversity in Chordate Evolution.

[12] Herrel A., Podos J., Huber S.K., Hendry A.P. (2005). Evolution of bite force in Darwin’s finches: A key role for head width. J. Evol. Biol..

[13] Barros F.C., Herrel A., Kohlsdorf T. (2011). Head shape evolution in Gymnophthalmidae: Does habitat use constrain the evolution of cranial design in fossorial lizards?. J. Evol. Biol..

[14] Dumont E.R., Dávalos L.M., Goldberg A., Santana S.E., Rex K., Voigt C.C. (2012). Morphological innovation, diversification and invasion of a new adaptive zone. Proc. R. Soc. Lond. B Biol. Sci..

[15] Openshaw G.H., Keogh J.S. (2014). Head shape evolution in monitor lizards (Varanus): Interactions between extreme size disparity, phylogeny and ecology. J. Evol. Biol..

[16] Segall M., Cornette R., Godoy-Diana R., Herrel A. (2020). Exploring the functional meaning of head shape disparity in aquatic snakes. Ecol. Evol..

[17] Adams D.C. (2011). Quantitative genetics and evolution of head shape in *Plethodon* Salamanders. Evol. Biol..

[18] Klingenberg C.P., Leamy L.J. (2001). Quantitative genetics of geometric shape in the mouse mandible. Evolution.

[19] Gmel A.I., Burren A., Neuditschko M. (2022). Estimates of genetic parameters for shape space data in franches-montagnes horses. Animals.

[20] Martínez-Abadías N., Esparza M., Sjøvold T., González-José R., Santos M., Hernández M. (2009). Heritability of human cranial dimensions: Comparing the evolvability of different cranial regions. J. Anat..

[21] Cole J.B., Manyama M., Larson J.R., Liberton D.K., Ferrara T.M., Riccardi S.L., Li M., Mio W., Klein O.D., Santorico S.A. (2017). Human facial shape and size heritability and genetic correlations. Genetics.

[22] King R.B. (1997). Variation in brown snake (*Storeria dekayi*) morphology and scalation: Sex, family, and microgeographic differences. J. Herpetol..

[23] Lucchini N., Kaliontzopoulou A., Val G.A., Martínez-Freiría F. (2020). Sources of intraspecific morphological variation in *Vipera seoanei*: Allometry, sex, and colour phenotype. Amphib. Reptil..

[24] Shine R. (1994). Sexual size dimorphism in snakes revisited. Copeia.

[25] Bonnet X., Shine R., Naulleau G., Vacher-Vallas M. (1998). Sexual dimorphism in snakes: Different reproductive roles favour different body plans. Proc. R. Soc. Lond. B Biol. Sci..

[26] Meik J.M., Setser K., Mociño-Deloya E., Lawing A.M. (2012). Sexual differences in head form and diet in a population of Mexican lance-headed rattlesnakes, *Crotalus polystictus*. Biol. J. Linn. Soc. Lond..

[27] Schwenk K. (2000). Feeding: Form, Function, and Evolution in Tetrapod Vertebrates.

[28] Da Silva F.O., Fabre A.C., Savriama Y., Ollonen J., Mahlow K., Herrel A., Müller J., Di-Poï N. (2018). The ecological origins of snakes as revealed by skull evolution. Nat. Commun..

[29] Gopalakrishnakone P. (1994). Sea Snake Toxinology.

[30] Brodie E.D. (1993). Homogeneity of the genetic variance-covariance matrix for antipredator traits in two natural populations of the garter snake *Thamnophis ordinoides*. Evolution.

[31] Westphal M.F., Massie J.L., Bronkema J.M., Smith B.E., Morgan T.J. (2011). Heritable variation in garter snake color patterns in postglacial populations. PLoS ONE.

[32] Martínez-Freiría F., Santos X. (2015). Assessing the heritability of dorsal pattern shape in *Vipera latastei*. Amphib. Reptil..

[33] Brown G.P., Shine R. (2016). Maternal body size influences offspring immune configuration in an oviparous snake. R. Soc. Open. Sci..

[34] Levine B.A., Douglas M.R., Adams A.A.Y., Lardner B., Reed R.N., Savidge J.A., Douglas M.E. (2021). Trait heritability and its implications for the management of an invasive vertebrate. Biol. Invasions.

[35] Üveges B., Halpern B., Péchy T., Posta J., Komlósi I. (2012). Characteristics and heritability analysis of head scales of the Hungarian Meadow Viper *(Vipera ursinii rakosiensis*, Méhely 1893). Amphib. Reptil..

[36] Martínez-Castro A., Kaliontzopoulou A., Freitas I., Martínez-Freiría F. (2021). Macroevolutionary variation and environmental correlates of scalation traits in Eurasian vipers (Serpentes: Viperinae). Biol. J. Linn. Soc. Lond..

[37] Zelditch M.L., Swiderski D.L., Sheets H.D., Fink W.L. (2004). Geometric Morphometrics for Biologists.

[38] Adams D.C., Rohlf F.J., Slice D.E. (2013). A field comes of age: Geometric morphometrics in the 21st century. Hystrix.

[39] Joger U., Crnobrnja-Isailovíc J., Vogrin M., Corti C., Sterijovski B., Westerström A., Krecsák Á., Pérez-Mellado V., Sá-Sousa P., Cheylan M. (2009). Vipera ursinii: The IUCN Red List of Threatened Species.

[40] European Reptile & Amphibian Specialist Group (2019). Vipera ursinii ssp. rakosiensis: The IUCN Red List of Threatened Species.

[41] Halpern B. (2007). A Rákosi vipera LIFE-program. A Rákosi Vipera Védelme.

[42] Halpern B., Tóth C., Brankovits D., Péchy T., Major Á. (2010). A rákosi vipera (*Vipera ursinii rakosiensis*) tenyészprogram eredményei 2004 és 2009 között. Állattani közl..

[43] Péchy T., Halpern B., Sós E., Walzer C. (2015). Conservation of the hungarian meadow viper *Vipera ursinii rakosiensis*. Int. Zoo. Yearb..

[44] Rohlf F.J. (2006). tpsDig, version 2.05.

[45] Rohlf F.J. (1999). Shape statistics: Procrustes superimpositions and tangent spaces. J. Classif..

[46] Rohlf F.J., Slice D. (1990). Extensions of the Procrustes method for the optimal superimposition of landmarks. Syst. Zool..

[47] Baken E.K., Collyer M.L., Kaliontzopoulou A., Adams D.C. (2021). Geomorph v4.0 and gmShiny: Enhanced analytics and a new graphical interface for a comprehensive morphometric experience. Methods Ecol. Evol..

[48] Adams D.C., Collyer M.L., Kaliontzopoulou A., Baken E.K. (2021). Geomorph: Software for Geometric Morphometric Analyses. R Package Version 4.0.2. https://cran.r-project.org/package=geomorph.

[49] Gunz P., Mitteroecker P., Neubauer S., Weber G.W., Bookstein F.L. (2009). Principles for the virtual reconstruction of hominin crania. J. Hum. Evol..

[50] Klingenberg C.P., Barluenga M., Meyer A. (2002). Shape analysis of symmetric structures: Quantifying variation among individuals and asymmetry. Evolution.

[51] Collyer M.L., Adams D.C. (2019). Linear Model Evaluation with Randomized Residuals in a Permutation Procedure. R Package Version 0.4.0. https://CRAN.R-project.org/package=RRPP.

[52] Collyer M.L., Adams D.C. (2018). RRPP: An R package for fitting linear models to high-dimensional data using residual randomization. Methods Ecol. Evol..

[53] De Villemereuil P., Gimenez O., Doligez B. (2013). Comparing parent–offspring regression with frequentist and Bayesian animal models to estimate heritability in wild populations: A simulation study for Gaussian and binary traits. Methods Ecol. Evol..

[54] Wilson A.J., Réale D., Clements M.N., Morrissey M.M., Postma E., Walling C.A., Kruuk L.E.B., Nussey D.H. (2010). An ecologist’s guide to the animal model. J. Anim. Ecol..

[55] Lande R., Arnold S.J. (1983). The Measurement of selection on correlated characters. Evolution.

[56] Roff D. (2000). The evolution of the G matrix: Selection or drift?. Heredity.

[57] Klingenberg C.P., Debat V., Roff D.A. (2010). Quantitative genetics of shape in cricket wings: Developmental integration in a functional structure. Evolution.

[58] Myers E.M., Janzen F.J., Adams D.C., Tucker J.K. (2006). Quantitative genetics of plastron shape in slider turtles (*Trachemys scripta*). Evolution.

[59] Efimov V., Kovaleva V.Y., Markel A. (2004). A new approach to the study of genetic variability of complex characters. Heredity.

[60] Felice R.N., Randau M., Goswami A. (2018). A fly in a tube: Macroevolutionary expectations for integrated phenotypes. Evolution.

[61] Klingenberg C.P., Hallgrímsson B., Hall B.K. (2005). Developmental constraints, modules and evolvability. Variation: A Central Concept in Biology.

[62] Schluter D. (1996). Adaptive radiation along genetic lines of least resistance. Evolution.

[63] Hellmann J.J., Pineda-Krch M. (2007). Constraints and reinforcement on adaptation under climate change: Selection of genetically correlated traits. Biol. Conserv..

[64] Bégin M., Roff D.A. (2004). From micro to macroevolution through quantitative genetic variation: Positive evidence from field crickets. Evolution.

[65] Werner Y.L., Seifan T. (2006). Eye size in geckos: Asymmetry, allometry, sexual dimorphism, and behavioral correlates. J. Morphol..

[66] Hibbitts T.J., Fitzgerald L.A. (2005). Morphological and ecological convergence in two natricine snakes. Biol. J. Linn. Soc. Lond..

[67] Vörös J., Ursenbacher S., Jelíc D., Tomović L., Crnobrnja-Isailović J., Ajtić R., Sterijovski B., Zinenko O., Ghira I., Strugariu A. (2022). Well-known species, unexpected results: High genetic diversity in declining *Vipera ursinii* in central, eastern and southeastern europe. Amphib. Reptil..

[68] Tomović L., Carretero M.A., Ajtíc R., Crnobrnja-Isailovíc J. (2008). Evidence for post-natal instability of head scalation in the meadow viper (*Vipera ursinii*)—Patterns and taxonomic implications. Amphib. Reptil..

[69] Getabalew M., Alemneh T., Akeberegn D. (2019). Heritability and its use in animal breeding. Int. J. Vet. Sci. Technol..

[70] Lourdais O., Shine R., Bonnet X., Guillon M., Naulleau G. (2004). Climate affects embryonic development in a viviparous snake, *Vipera aspis*. Oikos.

[71] Ujvari B., Korsos Z., Pechy T. (2000). Life history, population characteristics and conservation of the Hungarian meadow viper (*Vipera ursinii rakosiensis*). Amphib. Reptil..

[72] Baron J.-P., Le Galliard J.-F., Ferrière R., Tully T. (2013). Intermittent breeding and the dynamics of resource allocation to reproduction, growth and survival. Funct. Ecol..

