# Supplementary material for: Head Shape Heritability in the Hungarian Meadow Viper Vipera ursinii rakosiensis
Source: Animals (Basel). 2023 Jan 16;13(2):322. doi: 10.3390/ani13020322 (PMC9854840; doi:10.3390/ani13020322)
Supplement: Supplementary file 1 [file animals-13-00322-s001.zip › animals-2116313-supplementary.pdf]

Table S1: Summary of the composition of the viper families analyzed.

Mother \ Father	1-b-01/01	1-bc-01/04	1-bc-02/04	1-fh-17/08	1-fh-28/09	1-gy-01/09
2-asx-07/00	7	0	0	0	0	0
2-b-01/01	30	0	14	0	0	0
2-bc-01/04	0	22	0	0	0	0
2-bc-04/04	0	0	9	0	0	0
2-fh-18/08	0	0	0	0	0	7
2-gy-02/09	0	0	0	6	0	9
2-s-24/01	11	0	0	0	0	0
2-td-03/06	0	0	0	4	13	0

Table S2: Results of (M)ANOVA comparisons implemented to test for sexual dimorphism in head shape and log(CS), in adults and offspring separately. df: degrees of freedom, SS: Sums of Squares, MS: Mean Squares, R^2: squared coefficient of association, F: corresponding F-value, Z: effect size, p: p-value derived through 1000 permutation cycles. Note that due to difficulties in identifying the sex of some newborn individuals, the sample size of offspring used to test for sexual dimorphism is lower than the total sample used for other analyses.

ADULTS								
shape		df	SS	MS	R^2	F	Z	p
	sex	1	$2.42*10^{-3}$	$2.42*10^{-3}$	0.04	0.46	-1.18	0.87
	Residuals	12	0.06	0.01	0.96			
	Total	13	0.07					
log(CS)		df	SS	MS	R^2	F	Z	p
	sex	1	$3.87*10^{-4}$	$3.87*10^{-4}$	0.01	0.10	-0.80	0.78
	Residuals	12	0.05	$3.90*10^{-3}$	0.99			
	Total	13	0.05					
OFFSPRING								
shape		df	SS	MS	R^2	F	Z	p
	sex	1	0.01	0.01	0.01	1.27	0.80	0.22
	Residuals	203	0.94	$4.61*10^{-3}$	0.99			
	Total	204	0.94					
log(CS)		df	SS	MS	R^2	F	Z	p
	sex	1	$1.10*10^{-4}$	$1.13*10^{-4}$	$1.20*10^{-4}$	0.02	-1.22	0.87
	Residuals	203	0.97	0.00	1.00			
	Total	204	0.97					
